# The role of prompt, voice, and personality factors in the acceptance and evaluation of AI-generated mindfulness exercises

**DOI:** 10.1038/s41598-025-21290-1

**Published:** 2025-10-07

**Authors:** Diel Alexander, Bäuerle Alexander, Teufel Martin, Jansen Christoph

**Affiliations:** 1 Clinic for Psychosomatic Medicine and Psychotherapy, LVR-University Hospital Essen, University of Duisburg-Essen, Essen, Germany; 2 Center for Translational Neuro- and Behavioral Sciences, University of Duisburg-Essen, Essen, Germany

**Keywords:** Meditation, Generative AI, Uncanny valley, e-mental health, Digital health, Human behaviour, Health care

## Abstract

AI-generated mindfulness exercises have the potential to provide tailored mindfulness interventions. However, the role of quality of AI-generated mindfulness exercises on their acceptance and evaluation is as of yet underexplored. The present work investigates effects of prompting (tailored versus non-tailored versus human), voice (trained versus non-trained versus human), and matching voice personality factors on the uncanniness, human likeness, and acceptance of AI-generated mindfulness exercises. In two experiments, n = 143 participants rated real and different variations of AI-generated mindfulness exercises. It was found that trained AI voices significantly improve the evaluation of AI-generated mindfulness exercises comparable to human controls, while no significant effects of prompting were found. A categorization task further showed that exercises by trained AI voices were indistinguishable from human mindfulness exercises. Furthermore, a mismatch of voice personality and setting (mindfulness) significantly decreased voice evaluation. The results demonstrate the importance of using trained and matching AI voices for the implementation of AI-generated mindfulness interventions.

**Supplementary Information:**

The online version contains supplementary material available at 10.1038/s41598-025-21290-1.

## Introduction

Mindfulness exercises are a day-to-day practice to improve mindfulness and well-being and are used in mental health interventions: mindfulness-based therapy (MBT) has been shown to reduce symptoms of various mental disorders^1–4^. One advantage of MBT is flexibility: the content and type of exercise can be tailored to the disorder and personalized to the individual^5^. However the creation or acquisition of tailored MBT exercises may be time- and resource-intensive, limiting the advantage of flexible MBT exercises. Improving the flexible generation of mindfulness exercises would ease a wide, low-threshold supply.

E-mental health (EMH) tools show various advantages for mental healthcare, for example by increasing efficiency through automatization^6^, by enhancing the general healthcare infrastructure^7^, or by improving decision making in diagnoses or treatment choice^8^. Artificial intelligence (AI) specifically has been implemented in mental health treatment in various ways, for example for the detection of mental health symptoms^9,10^, as conversational chatbots^11^, or to evaluate patient assignments^12^ and to analyse patient-therapist relationship^13^. One advantage of AI is the creation of treatment material via generative AI. One aspect of generative AI is the ability to generate content, such as mindfulness exercise texts, using large language models (LLMs) which may allow the efficient production of tailored MBT exercises. For example, meditation exercises can be enhanced through generative AI, for example through “wandering voices” following practitioners through the environment^14^. Generative AI is thus suitable for the efficient and flexible generation of tailored mindfulness exercises which would allow an improved supply of such exercises for the healthcare system.

Several studies investigated AI-based therapeutic apps (usually AI-driven conversation agents or chatbots) that included mindfulness trainings. Denecke et al.^15^ evaluated the intelligent chatbot SERMO designed for emotion regulation which includes mindfulness exercises in its CBT-based interventions. WYSA is a smartphone-based empathy-driven conversational AI that includes mindfulness exercises and that can improve mental health in clinical patients in multiple studies^16–18^. Other AI-based therapy tools with mindfulness exercises include YOUPER^19^, ChatPal^20^, and more^21–23^. Although digital mindfulness apps are becoming more common^24,25^, they lack variety and user-centred adaptability that could be overcome by AI-generated exercises^26^. There is relatively little exploration and evaluation of AI-generated mindfulness exercises^27–30^: LLMs have been used to generate meditation guidelines for robot-supported mindfulness practices^27^. Generative AI can further be used to create texts, audio, and visuals for guided meditation^28^. MindSpace is a noteworthy AI-based application promoting mindfulness skills via meditative journaling^29^. Another app, Ajivar, features an AI-driven mindfulness coach that has been successfully used to reduce mental health symptoms in college students during COVID-19^30^. The advantage of AI to create tailored mindfulness exercises based on tailored prompts has been noted consistently is research investigating AI-generated mindfulness exercises^27–30^. In this regard, prompting (i.e., formulating a specific request for a generative AI to generate the desired result) is essential for producing the tailored and personalized output^31^. Especially in the context of mindfulness exercises in creating exercises tailored or personalized exercises, the role of prompting is essential to optimally use its strength. However, there is little research on the role of tailored prompting in the context of AI-generated mindfulness exercises^28^. Thus, the advantage of tailoring for AI-generated MBT is yet unexplored.

Furthermore, research on the acceptance of AI-generated mindfulness exercises compared to natural exercises (e.g., audio records of a therapist’s meditation guidance) is mostly lacking. Hence, it is unclear whether AI-generated exercises are on par with treatment as usual. Another issue in the implementation of AI-generated mindfulness practices lies in the acceptability of the tasks. Synthetic voices may be perceived as eerie or strange^32^, reminiscent of the *uncanny valley* a phenomenon describing the negative evaluation of near humanlike entities^33,34^. Today’s AI-trained synthetic voices are able to overcome a vocal uncanny valley, while untrained synthetic voices may not^32^. Furthermore, Stein and MacDorman^35^ suggested that with increasing use of AI, a “new uncanny valley” may emerge concerning the perception of an artificial computer system possessing a mind; hence, an AI-generated voice may appear uncanny not because it deviates from human voices, but because it may impersonate them too well. AI-generated mindfulness exercises ought to overcome this uncanny valley of voices to be of viable use in clinical practice, yet the role of quality of AI-generated voices in the acceptance of AI-generated mindfulness exercises is yet to be investigated.

Adding characteristics to synthetic voices like personality or emotional state may increase their appeal: For example, female and extraverted voices are generally preferred^36^, but that people also prefer voices whose personalities match their own^37,38^. Furthermore, a mismatch of features within a stimulus may be a source of uncanniness^39^, including inappropriate actions in social situations^40^: an android that is otherwise not uncanny in appearance^41^ may appear eerie if its social behaviour is not appropriate to the context; specifically, an android not showing an appropriate facial expression was considered more eerie compare to the same android showing the expression. This “social uncanny valley” may also occur if an otherwise acceptable AI-generated voice does not match the situation, such as reading a mindfulness exercise. Similarly, MacDorman^42^ contrasts playwright Chikamatsu’s concepts of *conceptual realism* and *surface realism* in the context of the uncanny valley to predict that something may look realistic yet appear eerie due to a lack of coherence in a context and an actor’s intentions and emotions; in this sense, eeriness may result from an inconsistency of an actor’s perceived state in contrast to the situation. In the context of AI-generated mindfulness exercises, conceptual realism may be achieved by the coherence of the context (mindfulness exercise) and adequate attributions towards the speaker based on the tone of voice (e.g., a calm voice). It has been argued that arousal (physiological excitation) and valence (negative or positive subjective experience, sometimes as separate concepts) mark the fundamental properties of affective experiences^43^. For example, the circumplex model of affect^44^ orders affective states on those two dimensions. Calmness is associated with positive valence and a low state of arousal. A voice inappropriate for a mindfulness exercise may thus be a voice expressing a state of negative valence and/or high arousal, such as excitement.

The unified theory of acceptance and use of technology (UTAUT) model provides a well-established framework to measure the acceptance of adopting new technologies^45^. According to the UTAUT model, the intention to adopt a new technology is influenced by performance expectancy, effort expectancy, social influence, and facilitating conditions. A negative perception of AI-generated mindfulness exercises may reduce behavioural intention which would hinder the implementation of such exercises.

## Research question and hypotheses

The current work investigates the influence of text and voice factors on the acceptance of AI-generated mindfulness exercises in two experiments. In experiment 1, it is investigated whether 1 voice quality (trained vs untrained voices) and prompting (tailored vs untailored prompts) are evaluated similar to treatment-as-usual MBT exercises written and voiced by humans. In Given the cost-efficiency of AI-generated mindfulness exercises compared to treatment as usual variants, a non-inferiority comparison to human mindfulness exercises are sufficient. Evaluation is investigated via acceptance (UTAUT) and the uncanny valley framework.

- Hypothesis 1: Mindfulness exercises using AI-trained voices are perceived as less eerie, are accepted more, and are categorized as human more than mindfulness exercises using untrained AI voices (voice effect).
- Hypothesis 2: Mindfulness exercises generated via tailored prompts are perceived as less eerie, are accepted more, and are categorized as human more than mindfulness exercises generated via untailored prompts (tailoring effect).
- Hypothesis 3: Mindfulness exercises using AI-trained voices and generated via tailored prompts are more eerie, accepted, and categorized as humans as mindfulness exercises using human therapist voices and written by humans therapists (non-inferiority effect).

In experiment 2, according to the predicitons of the “social uncanny valley” and conceptual realism^40,42^, it is investigated whether the the appropriateness of the AI-trained voice (calm versus excited) affects the eeriness, realsm, and acceptance of AI-generated mindfulness exercises.

- Hypothesis 4: An inappropriate (excited) mindfulness exercise voice is perceived to be more eerie, less humanlike, and is less accepted compared to an appropriate (calm) mindfulness exercise voice.

## Methods

### Participants

For experiment 1, a power analysis with a small effect size of *d* = 0.25, *N* = 93 participants were necessary to reach a power of 1 minus *β* = 0.8. *N* = 93 participants were recruited online via Prolific, a participant recruitment website that provides high-quality recruitment^46^. A small effect size was chosen to detect even subtle potential differences in uncanniness as an approximation to analogously small yet significant effects (e.g., *d* = 0.26) have been observed in previous research on the uncanny valley in subtle differences for visual stimuli^47^. Participants, all German residents, were on average 31.49 years old (SD = 12.61), 43 were female and 50 male. For experiment 2, a power analysis with a medium effect size of *d* = 0.5, *N* = 41 participants were necessary to reach a power of 1—β = 0.8. Participants were recruited online via Prolific. A larger effect size compared to experiment 1 was chosen for experiment 2 to approximate medium effects observed on the uncanny valley of social behavior personality in a social situation in visual stimuli^40,48^. A total of *N* = 50 participants were recruited. Participants were all German residents on average 33.36 years old (SD = 10.97), 33 were male and 17 female. Participant samples from experiment 1 and 2 were different, and previous participation in experiment 1 was an exclusion criterion for participating in experiment 2 to eliminate confounding effects by repeated exposure to similar stimuli. Furthermore, German fluency was an inclusion criterion for both experiments as exercises were presented in German language.

### Stimuli

Mindfulness exercise stimuli were generated for six different conditions allowing the investigation of AI-generated text and voice features separately while comparing to human control stimuli. Voices were either spoken by a human (psychotherapist, used for treatment-as-usual mindfulness exercises), trained AI voices (generated via *elevenlabs* (www.elevenlabs.io); voices were trained using real human footage and adapted to express specific personalities; the voices *Emily* and *Dorothy* were chosen from the *elevenlabs* selection to match mindfulness exercises, as those voices had labels such as calm, pleasant, or meditation), and non-AI synthetic text-to-speech (TTS) voices created via a Legacy version of the TTS Google Cloud services. Texts were either written by a human (psychotherapist, used for treatment-as-usual mindfulness exercises), or generated by ChatGPT 3.5 using either tailored prompts or untailored prompts. For prompts used, see the Python code in the supplementary material (Listing A1).

In total, participants heard 18 voices, with three voices per condition. All recordings were cut to 30 s to ensure comparability.

*Human control* stimuli were voice recordings of a female voice actress delivering mindfulness interventions that are used for treatment-as-usual mindfulness interventions. The recordings consisted of exercises with different foci (e.g., movement and relaxation, smell, body sensation, creativity, positivity, experience of pain, stress management). A total of 16 recordings were used out of which three were randomly selected for each participants. The texts were created by licensed psychotherapists experienced with mindfulness (incl. AB, CJ, and MT) and voiced by a professional hired voice actress recorded in a sound studio. Stimuli are designed and used as part of mindfulness-based interventions for the treatment of patients. The stimuli are not publicly available.

*Human trained* stimuli were created by using transcriptions of the mindfulness texts from the *human control* condition. Voices were two AI-trained voices from eventlab.io. A total of 16 recordings were used out of which three were randomly selected for each participants.

*Tailored trained* stimuli were voices were AI-trained voices reading mindfulness exercises generated by ChatGPT 3.5 using tailored prompts. A total of 20 recordings were generated out of which three were randomly selected for each participants.

*Untailored trained* stimuli were created using mindfulness exercises generated using untailored prompts and read by AI-trained voices. A total of 20 recordings were generated out of which three were randomly selected for each participants.

*Tailored untrained* stimuli were mindfulness exercise texts generated via specific prompts and read by untrained AI-voices. A total of 20 recordings were generated out of which three were randomly selected for each participants.

Finally, *untailored untrained* voices were created using untailored mindfulness texts read by untrained AI-voices. A total of 20 recordings were generated out of which three were randomly selected for each participants.

For experiment 2, mindfulness exercise stimuli with appropriate (condition 1) and inappropriate (condition 2) voices were used. Stimuli from condition 1 were identical to the tailored-trained stimuli from Experiment 1. For inappropriate voices, two female voices (for comparison to the female voices in the first condition) with traits describing high arousal states were selected from elevenlabs (Gigi and Elli). Both voices read the same mindfulness exercise texts that are used in the first condition. Each voice read the same 20 texts. Hence, a total of 80 stimuli were created in total, consisting of 40 stimuli per condition with two voices per condition.

For an objective validation of arousal differences between voice conditions, the voices’ pitch and pitch variation (standard deviation) were compared between conditions as both are associated with the perception of higher voice arousal and high-arousal emotions^49,50^. The voice analysis software Praat (version 6.4.39) was used to extract average pitch and pitch variation using raw cross-correlation for voice analysis^51^. Calm voices (Emily and Dorothy) scored significantly lower pitch (*t*(59) = 9.91, *p* < 0.001, *d* = 2.22) and pitch variation (*t*(59) = 5.03, *p* < 0.001, *d* = 1.13) compared to the excited voices (Gigi and Elli), showing differences in objective voice characteristics indicative of voice arousal.

The prompt used to generate mindfulness exercises is shown in Listing A1 (supplementary material). All generated mindfulness texts as well as the human-created texts are publicly available (https://osf.io/tnxw7/).

### Measures

For each voice, participants rated voices on multiple scales taken from the uncanny valley index to measure uncanniness and human likeness^52^ and the UTAUT^45^. For uncanniness and human likeness, participants were asked to rate voices on the following semantic differential scales ranging from 0 to 100: boring–eerie, creepy–bland, mechanical–humanlike, and natural–artificial. For UTAUT, participants were asked three items on intention to use (“I would try such an online service”, “I would use such an online service if it were offered to me”, and “I would recommend such an online service to others”.). Finally, participants were asked to categorize the exercise on whether they think it is voices by a human or an AI. Measures were identical in both experiments.

### Procedure

Both experiments were conducted online. After providing electronic informed consent, participants filled out demographic questionnaires followed by the experiment. For both experiments, participants were instructed to be in a quiet, undisturbed environment for the duration of the experiment. Participants were requested to possess a functional audio system. Use of headphones was not required.

For experiment 1, each participant listened to and rated 18 recordings in a random order, with three randomly selected recordings per condition. For experiment 2, each participant listened to and rated 12 recordings in a random order, with six randomly selected recordings per condition (three per voice). Hence, participants heard 3 randomly selected recordings of the voices Elli (inappropriate), Gigi (inappropriate), Dorothy (appropriate), and Emily (appropriate) each in a random order.

For both experiments, participants rated each recording according to the measures described above and were able to restart recordings at any time.

### Data analysis

Data analysis was conducted via R (ver. 4.1). Linear mixed models with planned contrasts were used to investigate the hypotheses, using the package *lme4* and *lmerTest*^53^. Outliers, defined as 1.5 times interquartile range, were removed in experiment 1 for uncanniness (95), human likeness (28), and UTAUT acceptance (16), and in experiment 2 for uncanniness (17), human likeness (8), and UTAUT acceptance (6). For the uncanniness index, the item scales boring–eerie and creepy–bland were combined via averaging. For the human likeness index, the item scales mechanical–humanlike and natural–artificial were combined via averaging^52^. These two indexes were used for all further analysis.

## Results

### Experiment 1

Linear mixed models with stimulus type as a fixed factor and participant and base stimulus as random factors and random slopes reveal that stimulus type significantly predicted uncanniness (*F*(5,54) = 2.63, *p* = 0.034, *R*^2^_c_ = 0.55). P-adjusted post-hoc Tukey tests reveal that compared to the human-control voices, human-trained (*t*(1179) = 0.37, *p* > 0.999), tailored-trained (*t*(1179) = 0.15, *p* > 0.999), or untailored-trained voices (*t*(1179) = 1.22, *p* = 0.78). Meanwhile, tailored-trained voices were significantly less uncanny than tailored-untrained (*t*(1179) = 4.97, *p* < 0.001, *d* = 0.47) or untailored-untrained voices (*t*(1179) = 6.08, *p* < 0.001, *d* = 0.59). Furthermore, untailored-trained voices were less uncanny than untailored-untrained voices (*t*(1179) = 4.73, *p* < 0.001, *d* = 0.47), while untailored-untrained voices were not more uncanny than tailored-untrained voices (*t*(1179) = 1.17, *p* = 0.853).

**Fig. 1:**
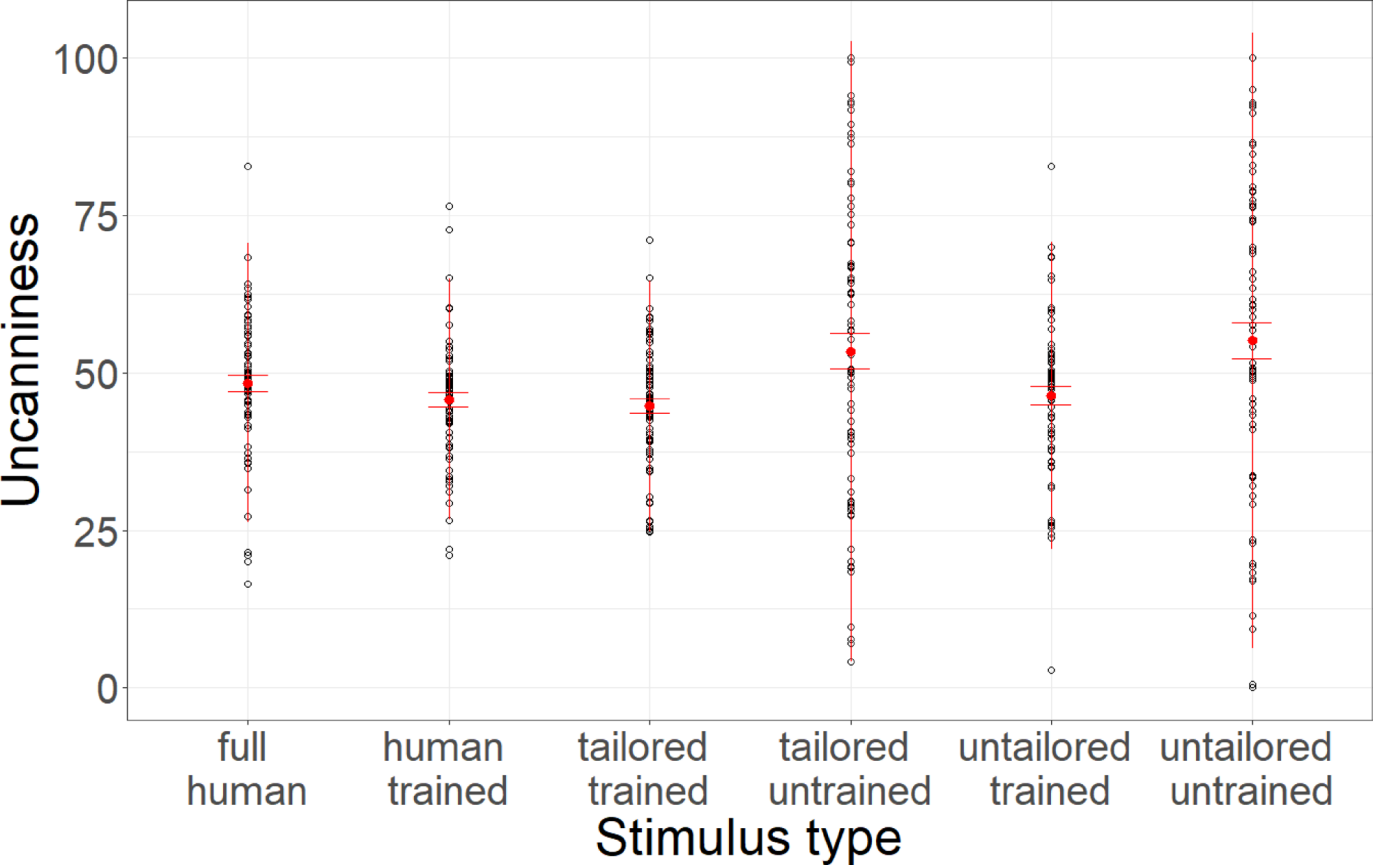
Uncanniness ratings. Average uncanniness ratings across stimulus conditions. Error bars indicate standard errors.

Additional comparisons further reveal that human-control voices were not more uncanny than the human-trained (*t*(1179) = 1.35, *p* = 0.265) and tailored-trained voices (*t*(1179) = 2.01, *p* = 0.067), and untailored-trained voices (*t*(1179) = 0.72, *p* = 0.705). Data is depicted in Fig. 1.

Linear mixed models with stimulus type as a fixed factor and participant and base stimulus as random factors and random slopes reveal that stimulus type significantly predicted human likeness (*F*(5,46) = 139.17, *p* < 0.001, *R*^2^_c_ = 0.73). P-adjusted post-hoc Tukey tests reveal that human-control voices were not more humanlike than human-trained (*t*(1179) = 3.24, *p* > 0.999), tailored-trained (*t*(1179) = 2.50, *p* > 0.999), or untailored-trained (*t*(1179) = 1.07, *p* > 0.999) voices. Meanwhile, tailored-trained voices were more humanlike than tailored-untrained (*t*(1179) = 26.71, *p* < 0.001, *d* = 2.56) and untailored-untrained voices (*t*(1197) = 27.66, *p* < 0.001, *d* = 2.68). Untailored-trained voices were more humanlike than untailored-untrained voices (*t*(1179) = 25.92, *p* < 0.001, *d* = 2.55), while tailored-untrained voices were not (*t*(1179) = 1.25, *p* = 0.74).

**Fig. 2:**
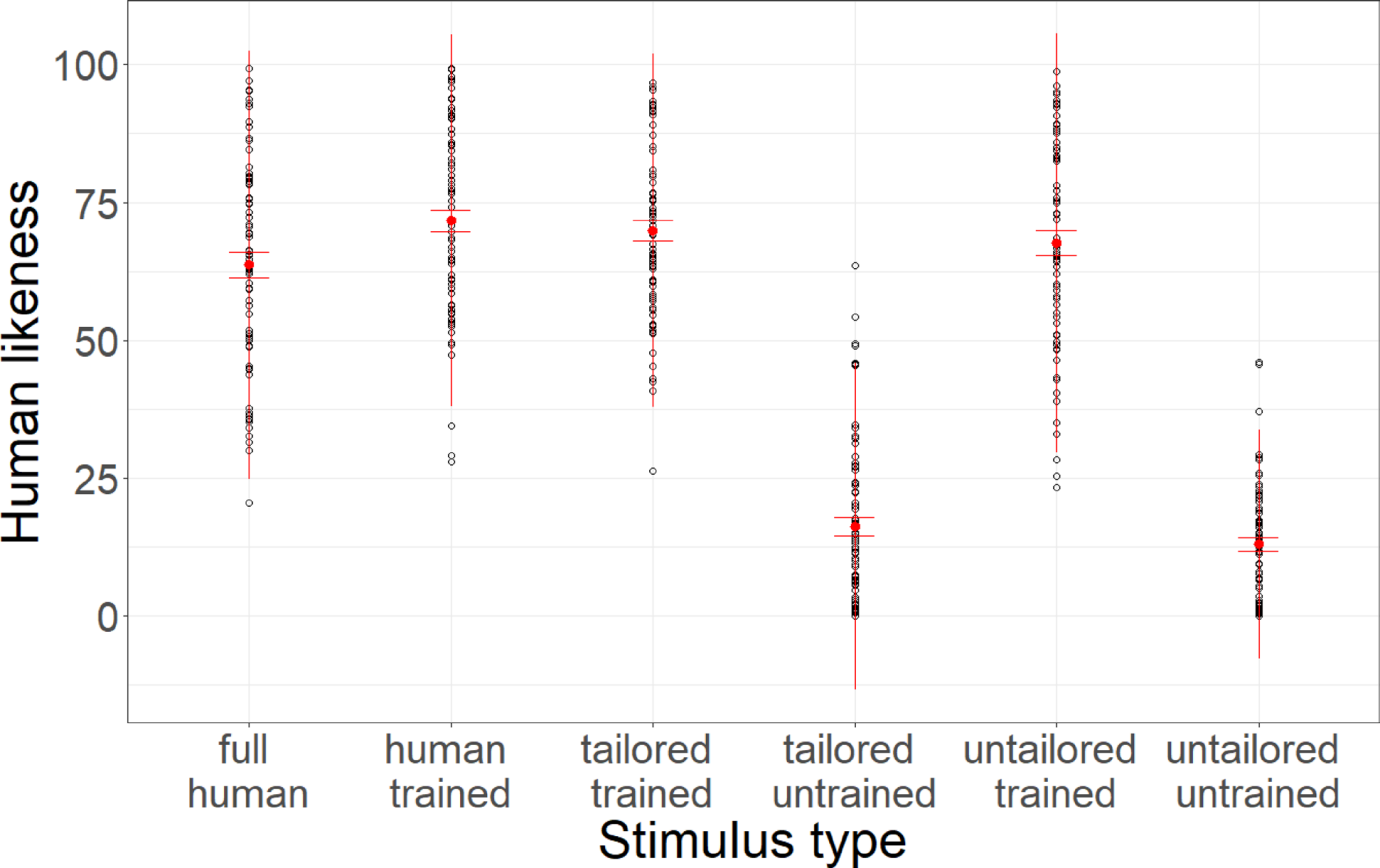
Human likeness ratings. Average human likeness ratings across stimulus conditions. Error bars indicate standard errors.

Additional comparisons further reveal that the human-trained (*t*(1179) = 3.24, *p* = 0.002, *d* = 0.32) and tailored-trained voices (*t*(1179) = 2.50, *p* = 0.019, *d* = 0.24) were perceived as more humanlike than the human-control voices, while the untailored-trained voices did not (*t*(1179) = 1.07, *p* = 0.429). Data is depicted in Fig. 2.

Linear mixed models with stimulus type as a fixed factor and participant and base stimulus as random factors and random slopes reveal that stimulus type significantly predicted UTAUT acceptance (*F*(5,54) = 35.47, *p* < 0.001, *R*^2^_c_ = 0.78). P-adjusted post-hoc Tukey tests reveal that acceptance of human-control voices was not higher than for human-trained (*t*(1179) = 0.40, *p* > 0.999), tailored-trained (*t*(1179) = 0.15, *p* > 0.999), or untailored-trained (*t*(1179) = 1.22, *p* = 0.78) voices. Meanwhile, tailored-trained voices received higher acceptance than tailored-untrained (*t*(1179) = 15.85, *p* < 0.001, *d* = 1.52) and untailored-untrained voices (*t*(1179) = 17.57, *p* < 0.001, *d* = 1.71). Untailored-trained voices received higher acceptance than untailored-untrained voices (*t*(1179) = 16.28, *p* < 0.001, *d* = 1.6) while tailored-untrained voices did not (*t*(1179) = 1.92, *p* = 0.194).

**Fig. 3:**
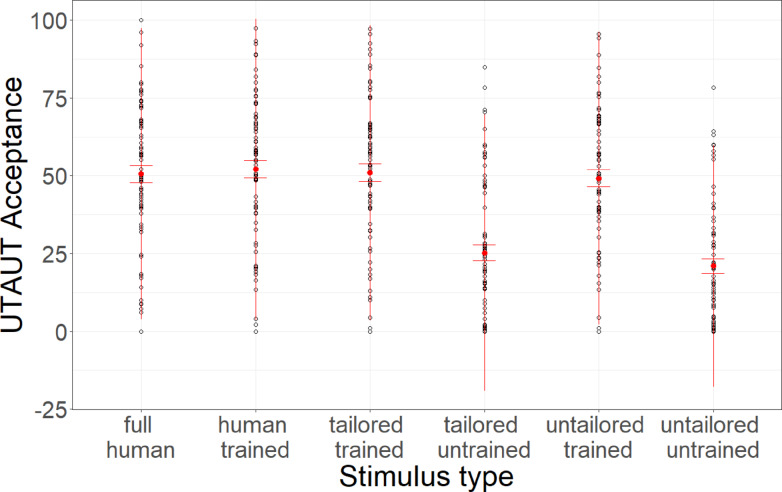
UTAUT acceptance ratings. Average UTAUT acceptances ratings across stimulus conditions. Error bars indicate standard errors.

Additional analyses revealed that compared to human-control voices, no higher acceptance was found for human-trained (*t*(1179) = 0.40, *p* > 0.999), tailored-trained (*t*(1179) = 0.15, *p* > 0.999), or untailored-trained (*t*(1179) = 1.22, *p* > 0.999) voices. Data is depicted in Fig. 3.

Finally, linear mixed models with stimulus type as a fixed factor and participant and base stimulus as random factors and random slopes reveal that stimulus type significantly predicted categorization as human or AI (*F*(5,42) = 104.86, *p* < 0.001, *R*^2^_c_ = 0.61). P-adjusted post-hoc Tukey tests reveal that human-control voices were not more likely to be categorized as human compared to human-trained (*t*(1179) = 2.49, *p* > 0.999), tailored-trained (*t*(1179) = 2.69, *p* > 0.999), or untailored-trained (*t*(1179) = 1.85, *p* > 0.999) voices. Meanwhile, tailored-trained voices were more likely to be categorized as human compared to tailored-untrained (*t*(1179) = 20.85, *p* < 0.001, *d* = 2.00) and untailored-untrained voices (*t*(1179) = 21.17, *p* < 0.001, *d* = 2.05). Untailored-trained voices were more likely to be categorized as human compared to untailored-untrained voices (*t*(1179) = 20.11, *p* < 0.001, *d* = 1.97) while tailored-untrained voices were not (*t*(1179) = 0.55, *p* > 0.999).

**Fig. 4:**
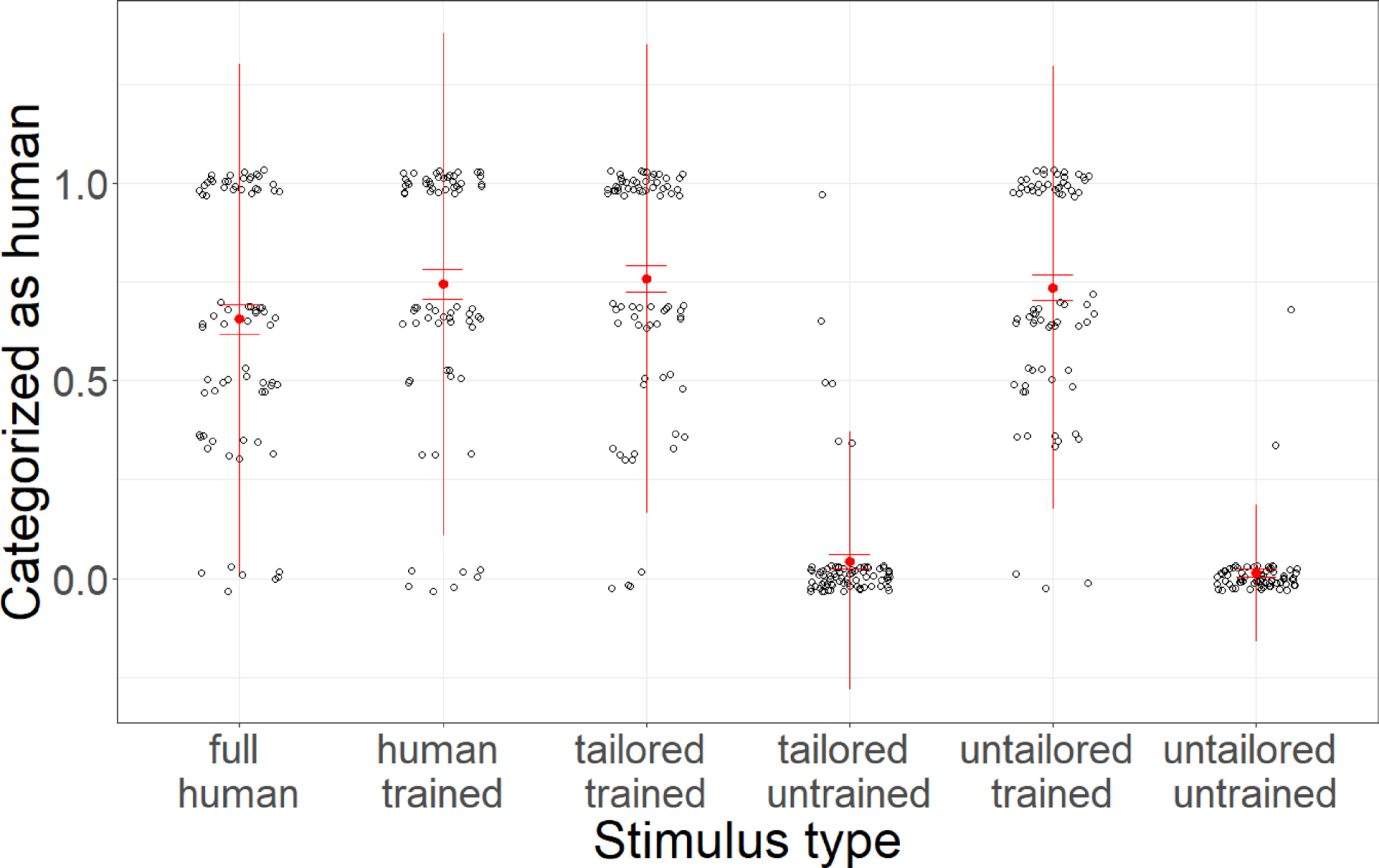
Categorization. Average categorization as human voices (versus AI voices) across stimulus conditions. Error bars indicate standard errors.

Finally, additional analyses revealed that compared to human-control voices, human-trained (*t*(1179) = 2.49, *p* = 0.019, *d* = 0.25) and tailored-trained (*t*(1179) = 2.69, *p* = 0.011, *d* = 0.26) voices were more likely to be categorized as human, whereas untailored-trained voices (*t*(1179) = 1.85, *p* = 0.098) were not. Data is depicted in Fig. 4.

An exploratory post-hoc analysis has been conducted investigating the role of participant age on voice evaluation depending on conditions. Linear mixed models were conducted investigating main and interaction effects of age on voice ratings. While no significant main effects of age were observed, results show a significant age-condition interaction only for uncanniness (*F*(5,111) = 2.70, *p* = 0.024, *R*^2^_c_ = 0.56). As can be seen in Figure A1 (supplementary material), younger participants tended to rate untrained voices as more uncanny compared to trained voices, while older participants did not.

## Experiment 2

**Fig. 5:**
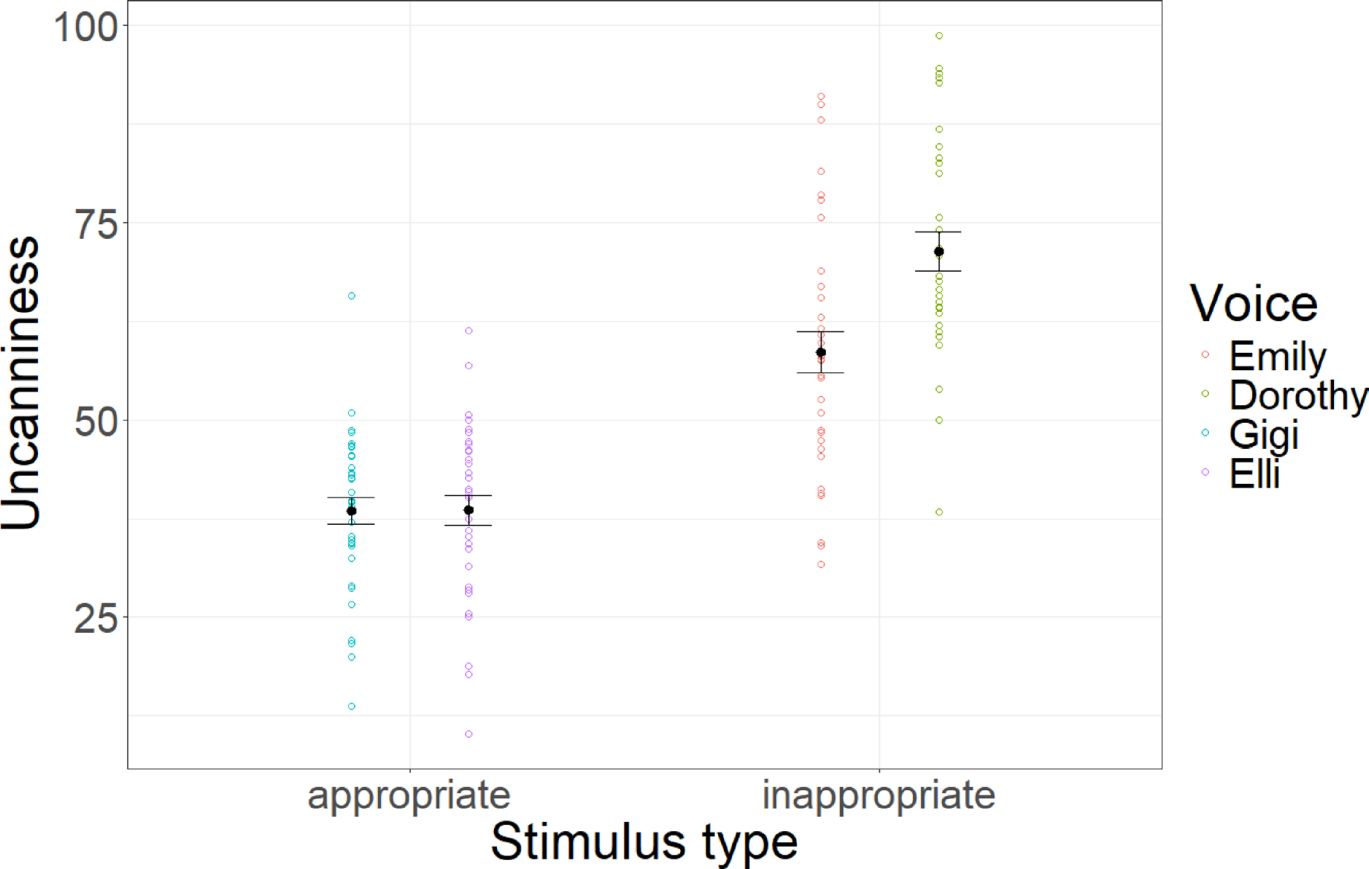
Uncanniness ratings. Average uncanniness ratings across stimulus conditions and voices. Error bars indicate standard errors.

Linear mixed models with condition as a fixed factor and participant and mindfulness text as random factors and random slopes reveal that condition significantly predicted eeriness (*F*(1,32) = 90.56, *p* < 0.001, *R*^2^_cor_ = 0.55). Controlled contrast shows that inappropriate voices were significantly more eerie than appropriate voices (*t*(368) = 10.22, *p* < 0.001, *d* = 1.71). Results are depicted in Fig. 5.

**Fig. 6:**
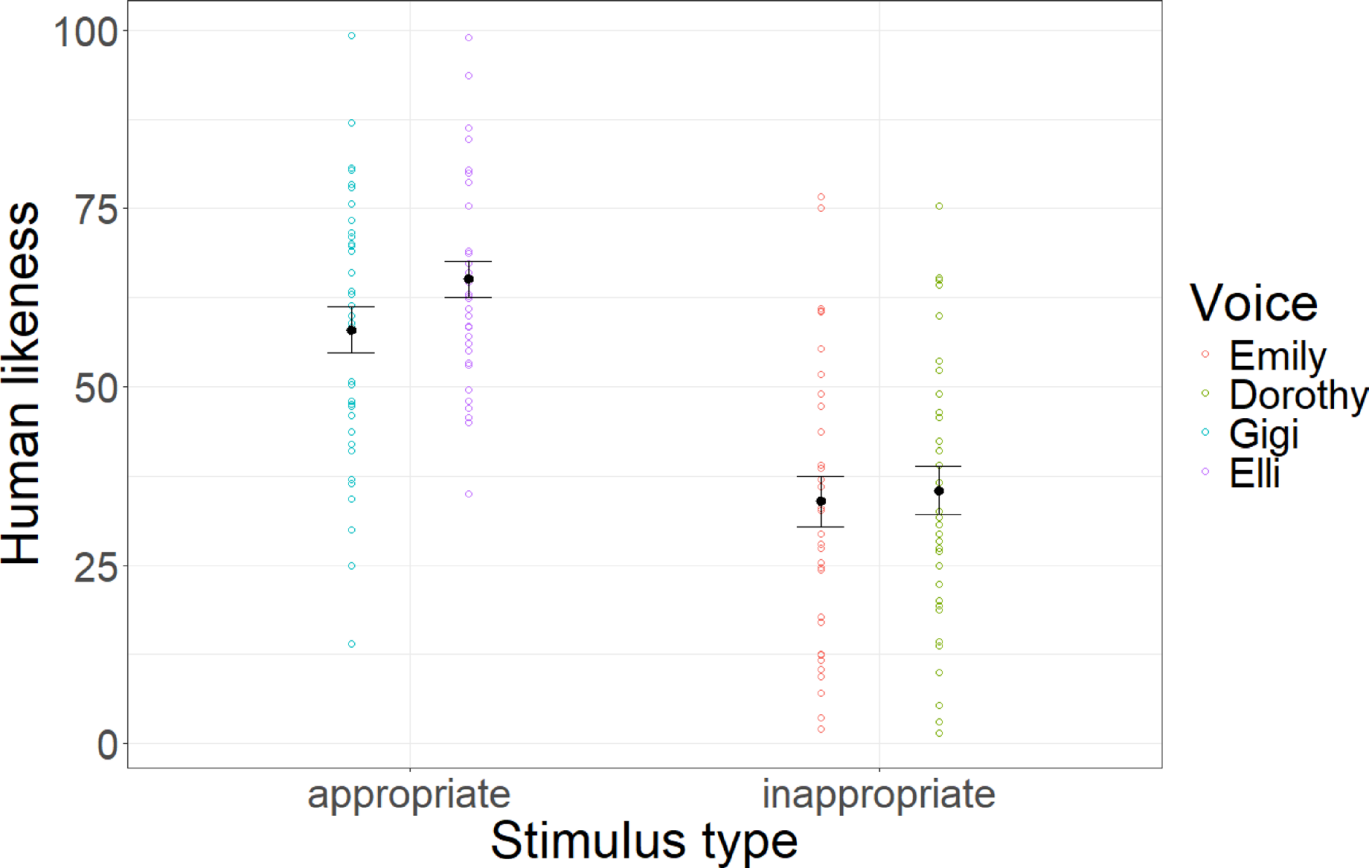
Human likeness ratings. Average human likeness ratings across stimulus conditions and voices. Error bars indicate standard errors.

Linear mixed models with condition as a fixed factor and participant and mindfulness text as random factors and random slopes reveal that condition significantly predicted human likeness (*F*(1,35) = 39.89, *p* < 0.001, *R*^2^_cor_ = 0.45). Controlled contrast shows that inappropriate voices were significantly more eerie than appropriate voices (*t*(368) = 11.221, *p* < 0.001, *d* = 1.12). Results are depicted in Fig. 6.

**Fig. 7:**
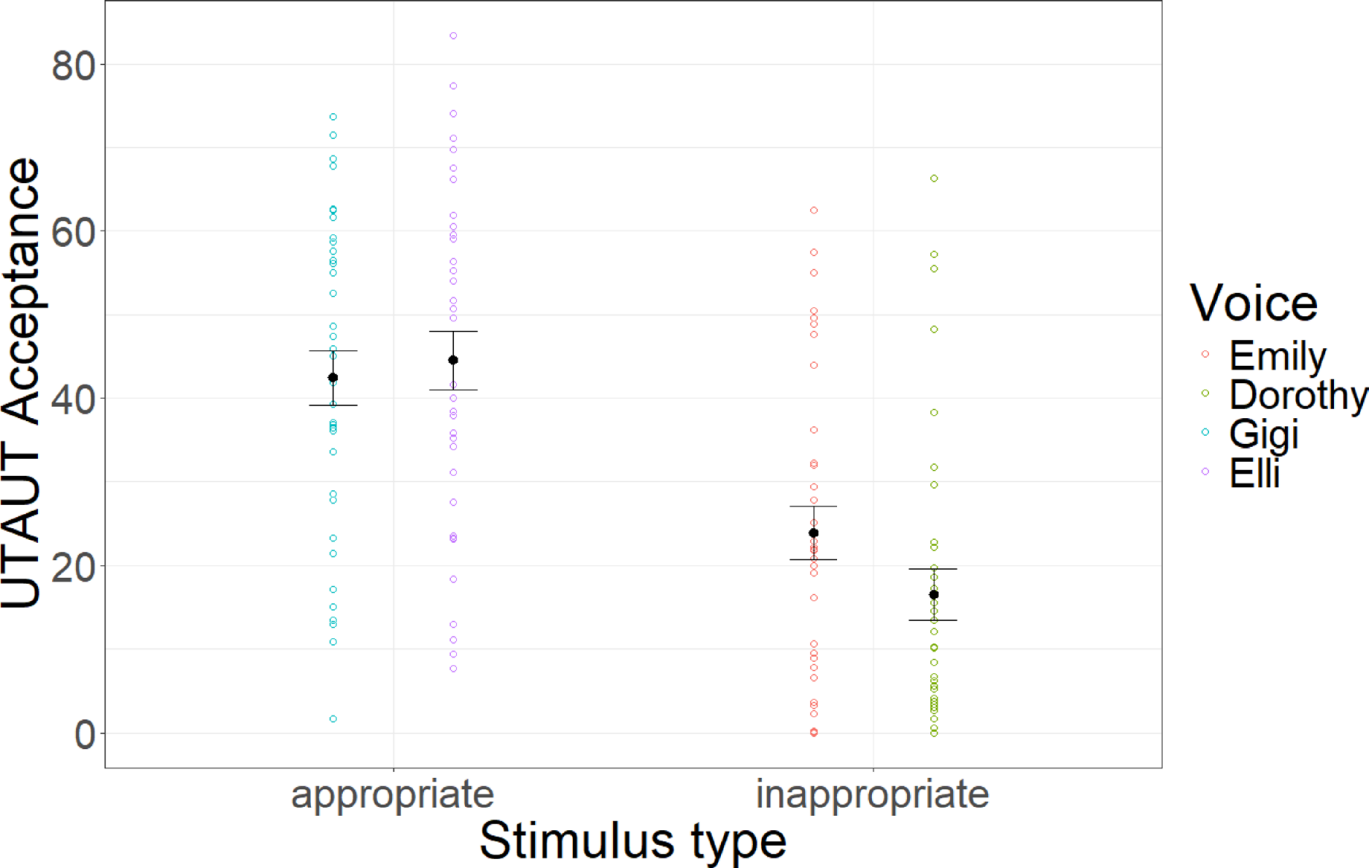
UTAUT acceptance. Average UTAUT acceptance ratings across stimulus conditions and voices. Error bars indicate standard errors.

Linear mixed models with condition as a fixed factor and participant and mindfulness text as random factors and random slopes reveal that condition significantly predicted UTAUT Acceptance (*F*(1,33) = 58.28, *p* < 0.001, *R*^2^_cor_ = 0.64). Controlled contrast shows that inappropriate voices were significantly more eerie than appropriate voices (*t*(368) = 13.65, *p* < 0.001, *d* = 1.36). Results are depicted in Fig. 7.

**Fig. 8:**
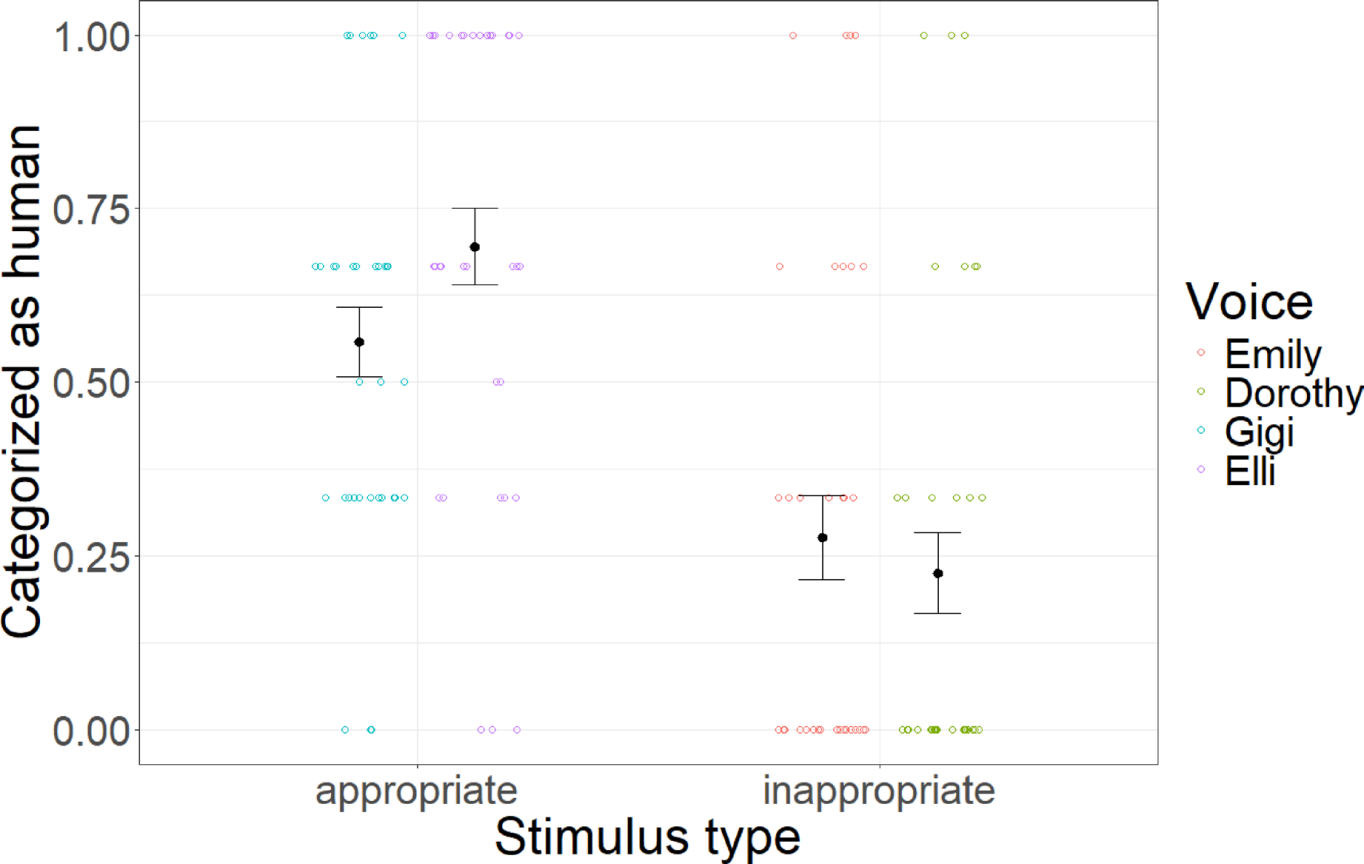
Categorization. Average categorizations as human (versus AI-generated) across stimulus conditions and voices. Error bars indicate standard errors.

Finally, linear mixed models with condition as a fixed factor and participant and mindfulness text as random factors and random slopes reveal that condition significantly predicted categorization as either human or AI-generated (*F*(1,31) = 33.27, *p* < 0.001, *R*^2^_cor_ = 0.33). Controlled contrast shows that inappropriate voices were significantly more eerie than appropriate voices (*t*(368) = 8.77, *p* < 0.001, *d* = 0.87). Results are depicted in Fig. 8.

An exploratory post-hoc analysis has been conducted investigating the role of participant age on voice evaluation depending on conditions. Linear mixed models were conducted investigating main and interaction effects of age on voice ratings. While no significant main effects of age were observed, results show a significant age-condition interaction only for UTAUT acceptance (*F*(1,33) = 6.23, *p* = 0.018, *R*^2^_c_ = 0.64). As can be seen in Figure A2 (supplementary material), older participants tended to rate appropriate voices as more acceptable compared to younger participants.

## Discussion

### Summary of results

AI-generated mindfulness exercises enable cost-efficient generation of exercises which are tailored to condition-specific and individual needs. The present experiments investigated the role of prompt and voice factors in the perception and evaluation of AI-generated mindfulness exercises. Voice factors, such as using an AI-trained voice (compared to an untrained AI voice) and the appropriateness of the AI voice’s personality, play a critical role in the positive evaluation and acceptance of mindfulness exercises. Meanwhile, the present study did not find evidence that the quality of prompting influenced evaluation and acceptance.

The use of AI-trained voices compared to untrained voices significantly reduced eeriness and improved human likeness, human categorization, and UTAUT acceptance, supporting hypothesis 1. Meanwhile, using tailored over untailored prompts for generating mindfulness exercises showed no significant improvements; hence, hypothesis 2 was not supported. Finally, in a non-inferiority comparison, AI-generated mindfulness exercises (specifically tailored trained exercises) were not more eerie or less humanlike, accepted, or categorized as human compared to treatment-as-usual human mindfulness exercises. Hence, hypothesis 3 is not supported, showing no evidence that AI-generated mindfulness exercises are inferior to human mindfulness exercises.

Thus, the current study supports the notion of using AI-generated mindfulness exercises in practice by providing evidence of their acceptance and ability to bridge the uncanny valley of voices similar to other trained AI-generated voices^32^. These results support previous research showing that more realistic synthetic voices are rated better than more mechanical voices, and similar to human voices^32,54,55^.

In experiment 2, results show that excited voices inappropriate for mindfulness exercises are perceived as more eerie and less humanlike, are less accepted, and less likely to be categorized as human compared to calm voices appropriate for mindfulness exercises. Hence, the social appropriateness of a voice may be necessary for its perception as humanlike and acceptable and to overcome an uncanny valley effect.

### AI hyperrealism

If not labelled as AI-generated, participants are even more likely to rate trained AI voices as human, similar to how AI-generated faces may be perceived as more trustworthy and authentic compared to real faces^56^. The notion of “AI hyperrealism” or a tendency to perceive AI-generated content as more realistic than real content has been discussed by Miller et al.^57^, who propose that AI-generated content contain unique perceptual features that are misidentified as indicators of real stimuli by human participants. Concordantly, participants unaware of AI-generated faces tend to use AI-typical features to misidentify AI-generated faces as real^58^, indicating that AI-generated content may be perceived as real due to exaggerating features of real counterparts. However, these findings contrast previous research not finding a hyperrealism effect for AI-generated voices^59^: AI-generated and real voices were rated on the same levels of realism. Potentially, the use of an application context in this study (mindfulness exercises) may slightly favour personality-congruency in voices for realism ratings, increasing the human likeness of the calm synthetic voices. Future research may investigate to what degree context (and voice-context congruency) affect realism ratings and hyperrealism effects in synthetic voices.

AI hyperrealism may be an advantage for AI-generated mindfulness exercises as it may increase effect sizes by exaggerating mindfulness-relevant features in text and voice. Meanwhile,

### Prompting

While prompting did not affect acceptance and evaluation of AI-generated exercises, it may still be important for the efficacy of exercises for mental health. One advantage of mindfulness exercises lies in its ability to be tailored to individual needs and preferences^5^ which can be extended to tailoring to mental disorders, stressors, or meditation preferences. Such an advantage can be further refined by an AI’s ability to quickly generate tailored mindfulness exercises. Future research may investigate the importance of specific prompting and tailoring of AI-generated mindfulness exercises for their efficacy to reduce distress or mental health symptoms.

### Voice-context congruency

The present results confirm the notions of conceptual realism and of a “social uncanny valley” effect stemming from inappropriate behaviour in a social situation even if the actor appears otherwise realistic and acceptable^40,42^. As artificial human actors such as androids or AI-generated voices find increasing use in society, their appropriateness in the context of the specific social situation becomes more relevant in their acceptance, for example in the implementation of digital mental health tools.

Although previous research found that extraverted voices are generally preferred^36^, further research appealed to a more nuanced take on voice personality factors, such as a match between voice and user personality^37,38^ or between a synthetic agent (in terms of visual appearance) and their voice^60^. The present results expand this nuance onto a voice-context match: While objective analyses showed that the calm AI voices had lower pitch and pitch variation (indicative of lower extraversion), these voices were also preferred in the given mindfulness context. Hence, synthetic voice personality preference also depends on the social or application context. Future research may aim to extend this observation onto different voice personalities and contexts.

### The role of age

Explorative analyses show that participant age affected the uncanniness and acceptance ratings of voices depending on condition: In experiment 1, younger participants tended to judge untrained voices as more uncanny compared to trained voices while older participants did not (Figure A1). These results reflect previous research on how voice artificiality negatively affects outcomes of young people compared to older people ^61^. Potentially, older participants are more used to more artificial or mechanical voices (e.g., untrained synthetic voices), decreasing their uncanniness compared to younger participants. Interestingly, however, the effect of an appropriate (vs inappropriate) voice on acceptance was stronger with increasing age in experiment 2 (Figure A2). Hence, older participants seem more sensitive to whether the voice matches the social context or application.

### Application

The current research adds onto previous literature on AI-driven mindfulness exercises^27–30^. AI-generated exercises may be delivered by a social robot to further autonomize mindfulness sessions, for example in group sessions. Such tools may be used in patient-present healthcare contexts that may otherwise suffer from staff shortage, such as in inpatient care. AI-driven mindfulness sessions can also be provided online, allowing the application for participants that may otherwise not be reached, such as during the COVID-19 pandemic^30^. Another potential application settings are patients currently on a waiting list for mental health treatment; waiting periods may span several months^62–67^ and are associated with increases in economic costs and symptom severity as well as reductions of adherence and elevation of dropout rates. However, digital support during the waiting period is currently sparse^68^. Providing digital AI-generated mindfulness exercises may support and increase adherence of pre-treatment waiting period patients and thus fill a critical gap in the healthcare system.

Despite initial promise in terms of acceptance and preference, there is currently little research of the efficacy of AI-generated mindfulness exercises^30^, especially in the context of mental health patients. Further research ought to investigate the efficacy of AI mindfulness tools in the context of clinical non-inferiority trials.

### Limitations and future research

Although validated self-report measures for uncanniness and acceptance have been used, no indirect measures such as behavioural or physiological correlates have been recorded. Some behavioural markers, such as dislike frequency or avoidance reactions have been associated with the uncanny valley^33^. Future research may further validate the findings by correlating subjective ratings with behavioural and physiological measures of uncanniness and acceptance.

The present study only focused on aspects of acceptance, namely measured via UTAUT and uncanniness. Hence, it remains an open question whether AI-generated mindfulness exercises show analogous efficacy compared to treatment as usual, for example towards improving mindfulness or alleviating distress.

Participants in this study were furthermore merely asked to listen to and rate the exercises. They were not asked to take part in them. Some observed effects, such as the role of a voice-context congruency, may be weaker (e.g., due to a shift in focus when conducting an exercise versus rating a voice) or stronger (e.g., due to greater immersion during the exercise) when participants take part in exercises. In addition, it is unclear whether these effects influence the effects of mindfulness exercises on well-being, distress, mindfulness states, or analogous outcome variables.. Future research should aim to replicate the results for conducted AI mindfulness exercises to validate the effects in a practical setting. In addition future research may investigate how these effects influence outcomes of mindfulness exercises.

In experiment 2, calm and excited voices were compared in the context of mindfulness exercises, with calm voices equating appropriate and excited voices equating inappropriate voices. However, the experiment lacked conditions with the appropriateness switched for the voice types in order to more fully argue for the role of mismatch on conceptual realism and uncanniness. Future research may investigate the role of voice-context matching in various different situations.

## Conclusion

AI-generated mindfulness exercises have the potential to become a flexible, tailored, and efficient tool to improve well-being. Despite initial promising findings, the factors influencing the acceptance and evaluation of AI-generated mindfulness exercises remain unclear. The present results indicate that the use of trained AI voices with matching personality is important to facilitate acceptance of AI-generated mindfulness exercises while being indistinguishable from human-led mindfulness exercises, although future is needed to investigate the effects in a practical setting. Hence, while current AI voices and texts are generally accepted, issues in their conceptual realism (e.g., voice-context match) may push them into an uncanny valley. The results pave a promising step towards the development and implementation of AI-generated mindfulness exercises for healthcare. Future research may focus on validating findings with behavioural or physiological measures, and by replicating results in clinical populations or when mindfulness exercises are conducted.

## Supplementary Information

Supplementary Material 1

## Data Availability

Data, analysis, and AI-generated exercises are publicly available at [https://osf.io/tnxw7/].
