# Supplementary material for: The role of prompt, voice, and personality factors in the acceptance and evaluation of AI-generated mindfulness exercises
Source: Sci Rep. 2025 Oct 7;15:35024. doi: 10.1038/s41598-025-21290-1 (PMC12504707; doi:10.1038/s41598-025-21290-1)

Listing A1. Python code used to generate tailored and untailored mindfulness exercise texts.

#Untailored:

{

"exercise_language": "German",

"communication_tone": "neutral",

"creation_instructions": "Create a mindfulness exercise focusing on the listener's body, using the informal 'Du' form to personally address the listener. The exercise should last approximately 30 seconds when read aloud."

}

#Tailored:

{

"exercise_language": "German",

"communication_tone": "neutral",

"creation_instructions": "Create a mindfulness exercise focusing on the listener's body, using the informal 'Du' form to personally address the listener. The exercise should last approximately 30 seconds when read aloud.",

"content_guidance": "The exercise should employ simple and direct questions that engage the sensory experience, with occasional prompts for the participant to reflect on their emotions. Aim to assist listeners in engaging mindfully with themselves, encouraging introspection on their reactions and emotions. The content should be accessible and inclusive, fostering a non-judgmental atmosphere and deeply immersive sensory experience. It's geared towards individuals new to mindfulness or those who have not previously engaged in such exercises, focusing on personal reflections related to body interactions, perceptions, and emotions."

}

Figure A1

Linear best-fit lines presenting the associations between participant age and uncanniness ratings across conditions (stimtype) for experiment 1. Grey areas indicate confidence intervals.


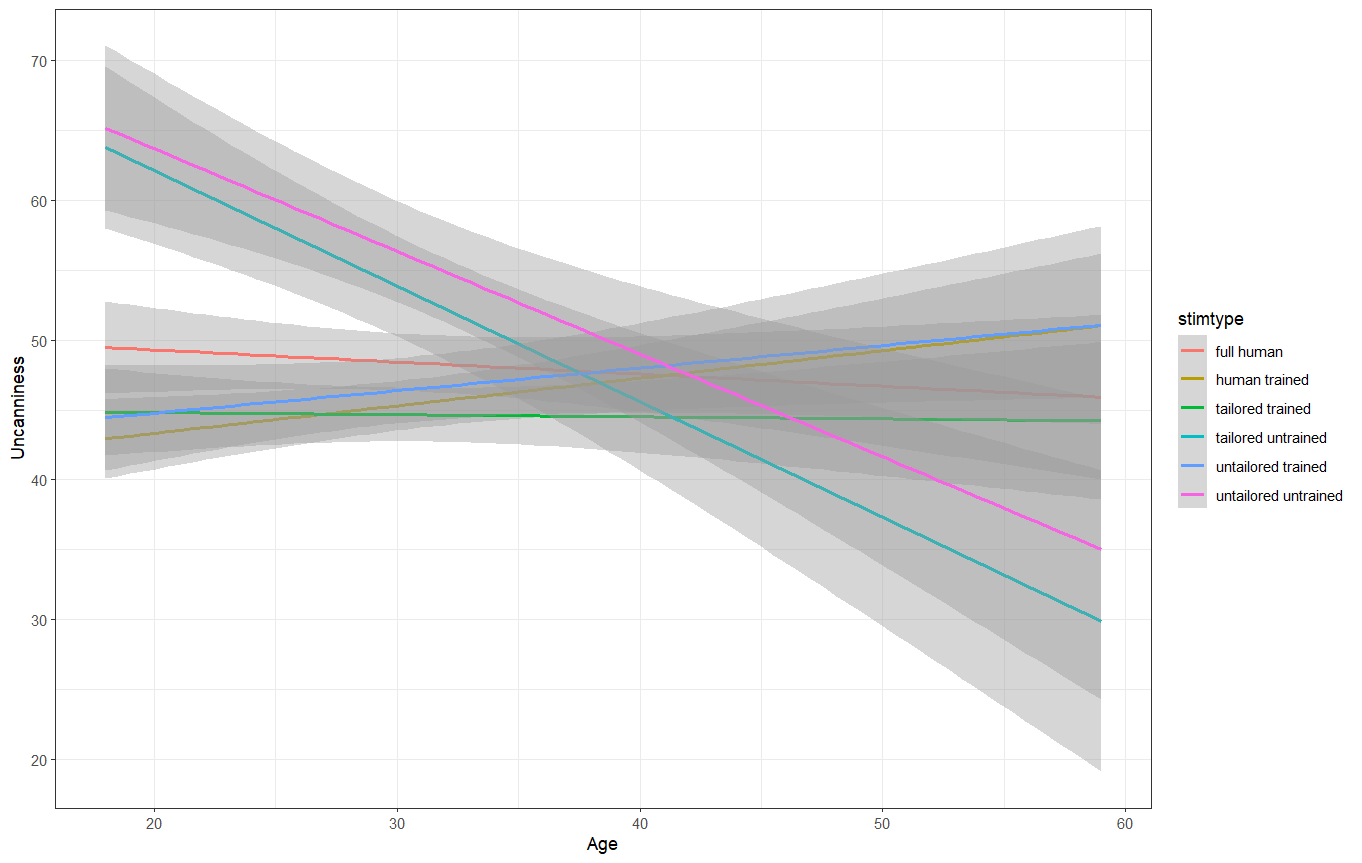


Figure A2.

Linear best-fit lines presenting the associations between participant age and uncanniness ratings across conditions (appropriate, inappropriate) for experiment 2. Grey areas indicate confidence intervals.


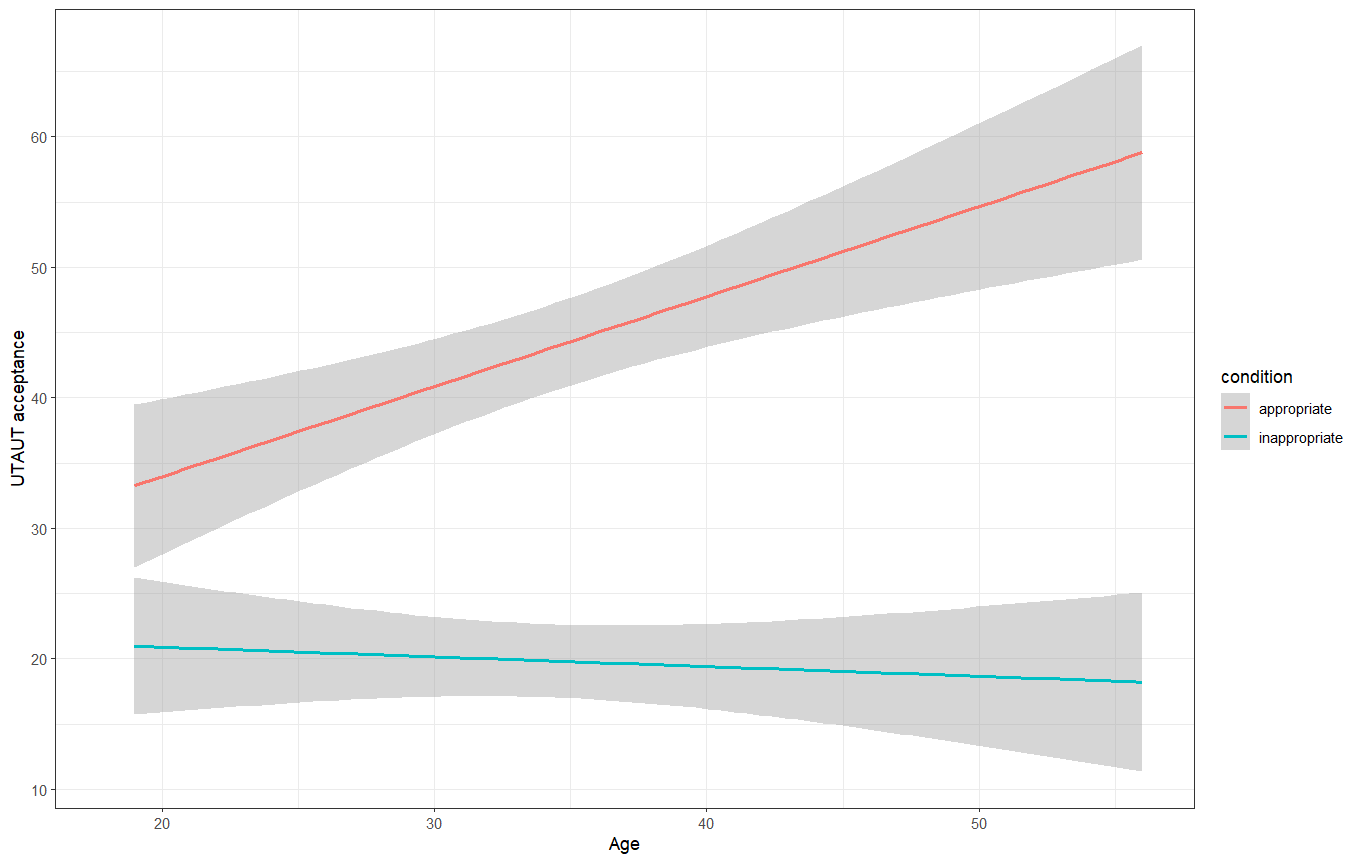

Supplement: Supplementary file 1 — Supplementary Material 1 [file 41598_2025_21290_MOESM1_ESM.docx]
